# Developing open-source software for bioimage analysis: opportunities and challenges

**DOI:** 10.12688/f1000research.52531.1

**Published:** 2021-04-19

**Authors:** Florian Levet, Anne E. Carpenter, Kevin W. Eliceiri, Anna Kreshuk, Peter Bankhead, Robert Haase

**Affiliations:** 1 Univ. Bordeaux, CNRS, Interdisciplinary Institute for Neuroscience, IINS, UMR 5297, Bordeaux, 33000, France; 2 Univ. Bordeaux, CNRS, INSERM, Bordeaux Imaging Center, BIC, UMS 3420, US 4, Bordeaux, 33000, France; 3 Imaging Platform, Broad Institute of MIT and Harvard, Cambridge, MA, USA; 4 Medical Physics and Biomedical Engineering, University of Wisconsin-Madison, Madison, WI, USA; 5 European Molecular Biology Laboratory, Heidelberg, Germany; 6 Pathology, Institute of Genetics and Molecular Medicine, University of Edinburgh, Edinburgh, UK; 7 DFG Cluster of Excellence “Physics of Life”, TU Dresden, Dresden, Germany

**Keywords:** Open-source, software, bioimage analysis, life science

## Abstract

Fast-paced innovations in imaging have resulted in single systems producing exponential amounts of data to be analyzed. Computational methods developed in computer science labs have proven to be crucial for analyzing these data in an unbiased and efficient manner, reaching a prominent role in most microscopy studies. Still, their use usually requires expertise in bioimage analysis, and their accessibility for life scientists has therefore become a bottleneck.

Open-source software for bioimage analysis has developed to disseminate these computational methods to a wider audience, and to life scientists in particular. In recent years, the influence of many open-source tools has grown tremendously, helping tens of thousands of life scientists in the process. As creators of successful open-source bioimage analysis software, we here discuss the motivations that can initiate development of a new tool, the common challenges faced, and the characteristics required for achieving success.

## Introduction

Modern imaging techniques ^1– 5^ have brought microscopy into a computationally-intensive era by capturing biological systems at a level of spatial and temporal resolution never achieved before. ^6^ A modern imaging system can easily generate terabytes of data for a single acquisition, and it is not uncommon for a biological study to involve dozens of samples from several different conditions. This massive amount of data has driven the adoption of new computational methods at all steps involved in imaging, whether it be for image acquisition, ^7, 8^ restoration, ^9^ data storage ^10^ or real-time processing. ^11^ Bioimage analysis has therefore become a crucial step integrating data science techniques into life science. Yet, the multiplicity of computational solutions has shifted the bottleneck: many of these solutions require substantial computational expertise and customization, limiting their use among many life scientists.

This expertise issue has been partly addressed by the emergence of specialists known as bioimage analysts. Originating from diverse backgrounds, such as computer science, microscopy and life sciences, they exhibit an overall understanding of many steps involved in life science experiments, spanning from biology to microscopy and analysis. ^12^ Acting as bridge-builders between life scientists and computer scientists, bioimage analysts develop skills to identify and adapt the best available computational solutions to a specific biological problem. Often employed in core facilities, their task is to deliver tools and workflows to end users - typically in biology or biophysics oriented labs - which go beyond service provision into experimentation and innovative technology development. ^13^ Depending on the analysis task, a workflow can require several tools, often split among multiple software libraries and even programming languages, to be identified, combined, customized and optimized to properly address a specific scientific question. However, many biologists still do not have access to core facilities employing such bioimage analysts and lack the in-depth knowledge, experience, and time required to develop, validate and apply such sophisticated workflows.

Thus, an important challenge is to directly provide life scientists with comprehensive, fully-featured and easy-to-use tools for domain-specific quantitative measurements that they can execute themselves. This development effort has been taken on by a subset of bioimage analysts, usually computer scientists by training, who develop open-source software platforms to improve the accessibility of computational methods for both “power users” and biologists with very little computational training. The resulting open source image analysis platforms have achieved great success and had a long-lasting impact on life science: the ability to access analysis methods’ source code have accelerated their adoption, improved reproducibility and facilitated the combination and implementation of new methods. Nevertheless, anyone seeking to develop biology-oriented open-source software platforms faces numerous challenges in terms of packaging, documentation, maintenance, reproducibility and funding. In this opinion paper, we intend to discuss these aspects in a way that will be beneficial both for developers and users. Developers will be exposed to minimally required motivations that justify the creation of a new open-source tool, as well as best practices to follow during development. Users will be introduced to some of the challenges faced by developers, hopefully leading to a better understanding of the open-source bioimage analysis ecosystem, and how they might personally contribute to the development and support of the software tools they need.

## Choosing the software’s scope and audience

The development of a new bioimage analysis software platform is often motivated by the desire to answer a specific unmet biological question. However, bringing a broadly useful tool to users is a substantial commitment that typically requires many person-years of work ( Table 1). It is important that this is undertaken with an understanding of the specific challenges and opportunities, to reduce the risk of a promising project later being abandoned and leaving users unsupported. A crucial first question is to define the scope and intended audience for the software.

**Table 1.** Size, impact and timeline of a selection of open-source tools.

	Type	Total lines of code	Commits in 2020	Citations in 2020	Development time since project started (in months)	Timeline (start-end)
**JACoP** ^15^	Plugin	2,400	3	358	8	[2005-ongoing]
**SR-Tesseler/** **PoCA**	Software	100,800	1	56	75	[2012-ongoing]
**clij/clij2/** **assistant**	Library	100,000	2,500	12	20	[2018-ongoing]
**QuPath**	Software	110,000	570	655	60	[2016-ongoing]
**ilastik**	Software	155,000	910	442	200	[2011-ongoing]
**CellProfiler**	Software	280,770	492	1,740	216	[2003-ongoing]
**Bio-Formats**	Library	1,502,214	573	245	180	[2006-ongoing]
**ImageJ/FIJI**	Software	2,024,516	2,934	44,400	432	[1997-ongoing]
**OMERO**	Software	2,171,241	3,667	361	420	[2003-ongoing]
**IDR**	Repository	16,517,904	1,756	76	180	[2016-ongoing]

At one end of the spectrum, one can simply write the minimum code necessary to address a biological question, and provide scripts alongside the paper publishing the results. This is a perfectly acceptable route and should be considered for cases where the image analysis solution is heavily customized to the problem and thus of limited use to others, or where there is no strong prospects of future support, maintenance, and development. We should applaud efforts to still make code available, for reproducibility reasons, even when it is neither polished nor broadly useful. ^14^

**Figure 1.**
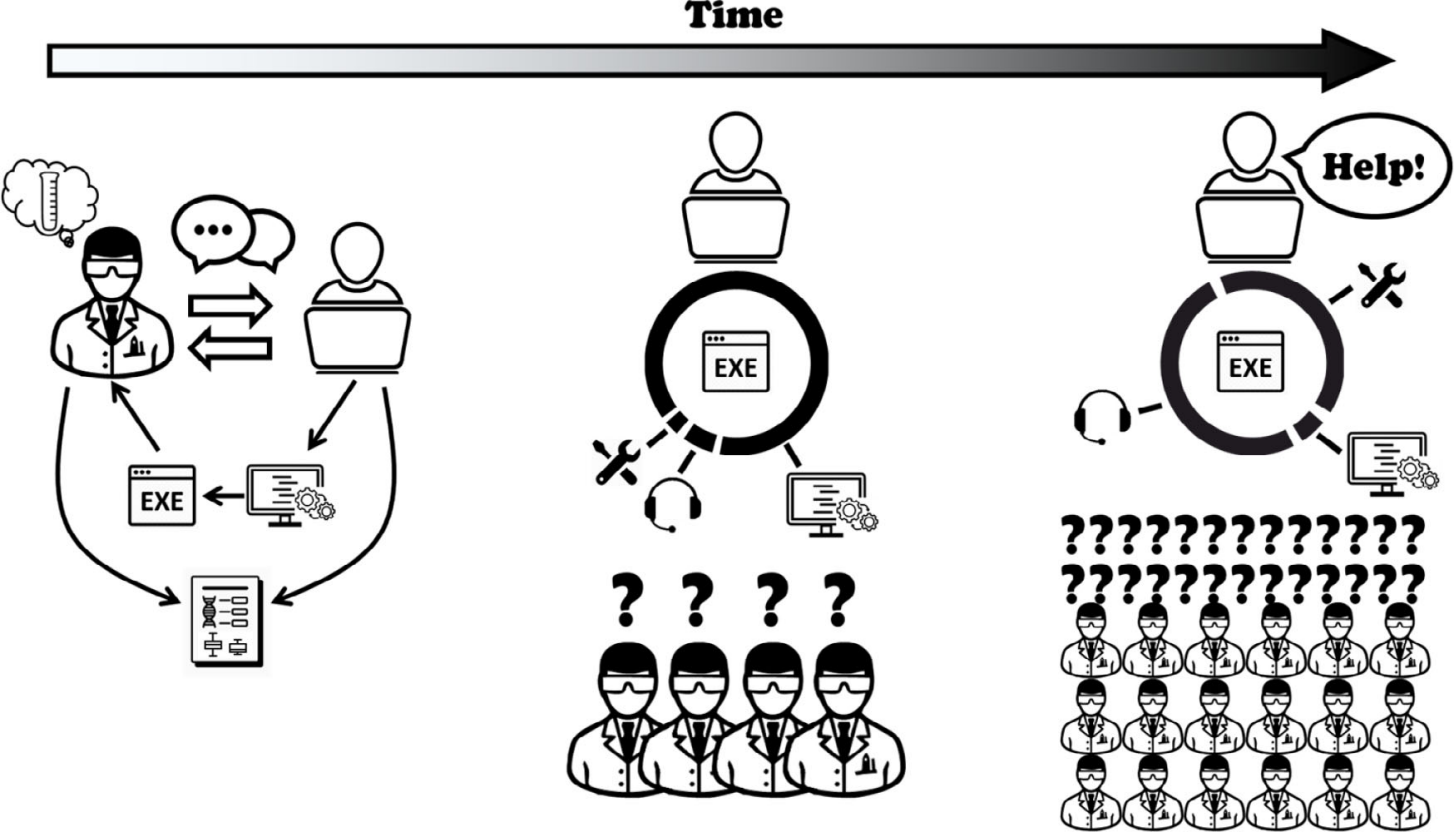
Lifetime of an open-source software. Created to solve a biological analysis need, the tool is released and published. As it is adopted by new users, the developer begins to spend time on user support and maintenance, while still managing to add new methods. Finally, as the number of users continues to grow, the developer is overwhelmed with user support and maintenance. As this stage, securing funding for new dedicated developers is crucial.

At the other end of the spectrum, one can launch an entirely new software effort around the code needed for a particular project, expanding this to serve a broader audience over time. Here, a crucial consideration is whether there is a plausible strategy for future support, maintenance, and development in the long term, especially if the software is widely adopted and therefore becomes relied upon by a community of scientists. A successful project may grow far beyond the initial expectations of the creator, and as this happens the nature of the required work tends to change ( Figure 1). In the words of Kurt Vonnegut, “[a] nother flaw in the human character is that everybody wants to build and nobody wants to do maintenance” - or, perhaps more relevant in this context, nobody wants to *fund* maintenance. ^16^

An intermediate position is to develop a plugin or extension for an existing platform. Extensibility has been key to the success of software projects such as ImageJ, ^17^ Fiji, ^18^ Icy ^19^ and CellProfiler, ^20^ with the result that many novel algorithms are developed as plugins for these platforms. One example is the CLIJ library, which brings manifold implementations of accelerated image processing algorithms as plugins to platforms such as ImageJ and Icy. ^11^ This strategy not only reduces effort for the developer, who can focus primarily on developing new functionality beyond that already available, but also reduces the effort required by users to become familiar with entirely new softwares. For this reason, we advocate developing plugins or adapting existing solutions wherever possible. This has the added benefit of improving the robustness of the core software: reusing components results in more thorough testing and therefore fewer undiscovered bugs.

Nevertheless, new open source bioimaging software applications are sometimes needed as the computational and biological landscape changes over time. If a major gap is identified, as was the case for supporting and visualizing multidimensional image data, ^21, 22^ then developing a new software platform can be the best way to serve the community and lead to great success. Indeed, if the software is user-friendly and well packaged, its adoption by the community can be swift. ^23– 28^

When launching a new software project, our experience is to carefully consider the boundaries of the audience to be served. As maintenance is an ongoing tax against future software developments (not to mention research progress, which is often necessary for funding the software), it is important to not be overly ambitious by attempting to create software that addresses too many distinct problems simultaneously. For example, in the CellProfiler project, we aimed to serve the community needing modular, automated pipelines, and left interactive manual analysis and image visualization tools to ImageJ, which was well-developed in these features. We also left a gap for very large-format images such as for pathology, which would have been difficult to support well given its underlying infrastructure; QuPath was later developed specifically to meet this need from the ground up. ^23^ Likewise, Single-Molecule Localization microscopy was not well served by generic bioimage analysis platforms, because it produces point clouds rather than conventional images; the Tesseler-suite ^25, 29^ and its successor PoCA ^30^ were created to meet this need. In another example, ilastik ^31^ was developed with the aim of providing interactive machine learning tools, intentionally leaving pre- and post-processing steps to Fiji and CellProfiler. OMERO has always remained largely focused on image storage and organization. ^32^ Defining the scope in this way prevents a project from being spread too thin and serving no audience very well. Furthermore, interoperability between software using open standards makes it possible to gain the benefits of combining applications.

**Figure 2.**
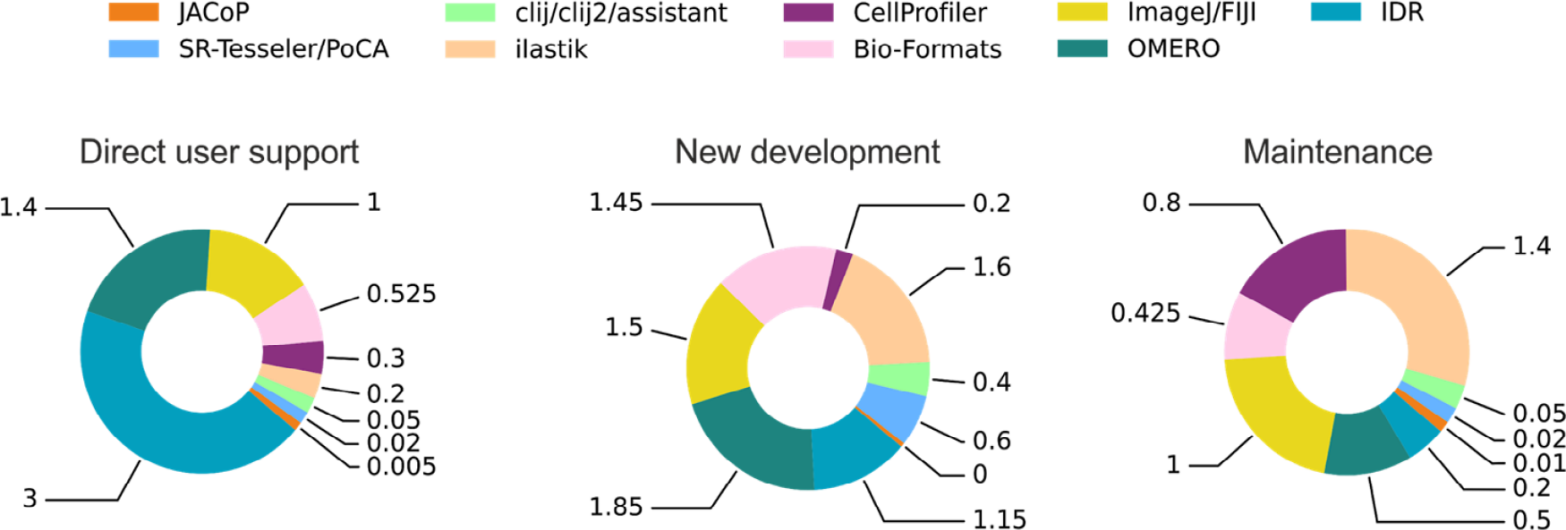
Developers' time (in person-units annually) devoted to user support, new development and maintenance for tools presented in Table 1. These charts only take into account paid staff developers.

Another choice to consider is whether to serve point-and-click users versus those who are comfortable writing code themselves; some software serves both ends of this spectrum well, but again defining the audience proactively ensures that developer time is not spread too thin meeting those needs. The skills and effort required to design an intuitive and responsive graphical user interface are quite distinct from those needed to devise new image analysis algorithms, and are therefore understandably not always a core focus. Furthermore, while a developer who chooses to concentrate on user-friendliness may find that their software is more widely adopted, this can be a double-edged sword in that it may dramatically increase demand for support, make delivering new functionality more onerous, and reduce the time available to focus on new projects or publications ( Figure 2). Some developers consider these costs justified because they can increase the impact of their work, but ultimately it is a decision that needs to be made: even highly-technical software is a creative endeavor through which developers can express their own interests and values.

## Programming language

The choice of programming language is a core decision that has long-term impact. It is usually a trade-off between the developer’s preference and experience versus external factors, such as an entity enforcing the use of a particular language to ensure compatibility between their tools, the need to use specific libraries, or the desire to interoperate with existing tools.

Traditionally, software platforms were usually developed in C/C++ or Java, but the last decade has seen the rapid emergence of Python in the scientific landscape. C/C++ can offer excellent performance and is still the dominant language in Computer Graphics, an important point when one wants to transfer this research to bioimage analysis tools, but portability can be an issue because tools developed in C/C++ need to be compiled for every targeted operating system. In contrast, Java applications are portable and can be executed on all major platforms, without the need for recompilation. Java applications can also readily use the image processing ecosystem around ImageJ ^17^ and Bio-Formats for image file reading/writing; ^33^ together these advantages help explain why so many bioimage analysis platforms have been developed in Java. Finally, recent years have seen an important increase in the number of workflows and tools developed in Python. Python has become the language of choice for data science and can be self-learned by non-computer scientists. Combined with its numerous available scientific libraries, Python represents a solution that is well-suited for developing new analysis methods using both image processing and machine learning techniques.

Each language has its pros and cons, and bridges exist to allow communication between tools developed in different languages. Nevertheless, working across programming languages makes development and distribution of software more difficult, introducing a challenge that can be hard to bear for small-sized research groups.

## Communities

Most bioimage analysis software platforms start with a single developer and a single user, who are often the same person. Over time many more users and developers can be attracted into a rich and healthy community. There are many ways to support open software that do not require writing code. Often, the most valuable contributions can be from experienced users providing examples, writing documentation, answering questions for their peers and creating online resources, and testing new release candidates.

In open source projects, the developer community can include individuals from all over the world who modify or develop new methods and ask the authorized maintainers to merge their work within the tool’s official code. This can be extremely beneficial, enhancing the software and broadening its scope. In widely used projects the maintainers can, however, become overwhelmed by requests, which risk pulling the project in different directions without dedicated engineers determining the future vision. There is also a risk that the creator of a successful application can feel reduced to an administrative role as their project grows, with their time being taken up by reviewing the contributions of others and answering user questions instead of solving problems and writing code themselves, with diminishing recognition for this essential work. Software designed for point-and-click users will by nature have fewer coding contributors than libraries designed for computationalists’ use. Ultimately, this shows the necessity for bioimage analysis tools to have both funded developers dedicated to handling the core software and plugin mechanisms allowing individuals to develop new methods without modifying the core code.

The bioimage analysis community is extremely welcoming and friendly. The Scientific Community Image Forum ( https://forum.image.sc/), ^34^ has rapidly emerged as an essential entry point in the field, and is the preferred place for any bioimage analysis related question. It is already the primary designated discussion point for more than 40 open source tools/libraries. One of its characteristics is that it is extremely easy for a new open-source bioimage analysis tool or library to be included as a community partner. Because of its wide audience, it can tremendously benefit developers by (i) facilitating the discovery and recognition of their tools and, (ii) alleviating the burden of answering new users' questions, both because other users of the software can pitch in and because answers remain publicly available and searchable. Finally, the forum creates a network of developers in which often-isolated developers can get help for technical issues.

## Packaging

Maximizing the impact of a new tool towards life scientists usually requires a well-designed Graphical User Interface (GUI) that allows users to interact with the software platform. As mentioned above, this can require considerable effort from the developer. Scientific image data are extremely variable, with the result that analysis often involves customization. It can be extremely challenging to provide a balance between user-friendliness, flexibility and extensibility within a GUI. A technical compromise can be to provide core functionality through a point-and-click interface supplemented by scripting for power users.

An easy and comprehensible installation process is just as important for adoption. For installation, traditionally, platforms developed in compiled languages (C/C++ or Java) are shipped with 1-click installers, the easiest solution possible, but requiring significant expertise from the developer. For the user, the best scenario is when all dependencies are directly included in the installer or are automatically downloaded. If they are not included, the user will have to install them separately, a potential source of issues and a support burden for the developer. Interpreted languages such as Python have slightly shifted this common process as scripts/apps can be bundled as packages and easily installed using package-management systems such as pip. In practice, dependencies or version issues are not uncommon, and installing Python modules may still require some level of expertise. Python scripts can also be bundled in web-based interactive computational environments (such as Jupyter Notebooks). To ensure simplicity, we advocate providing 1-click installers for bioimage analysis tools where feasible, supporting all three popular operating systems.

The time-consuming nature of packaging highlights another advantage of developing plugins for existing platforms: they already provide standardized GUI elements and installers that are familiar to users. Furthermore, if developers from different tools share efforts in improving and maintaining a pre-existing GUI, long-term adoption by a larger community can be achieved.

## Version control and quality assurance

The development process of scientific software includes two more important elements: Firstly, version control is good scientific practice, allowing developers to maintain their code in a modern fashion while tracing changes and contributions. The git platform ( https://git-scm.com/) is most commonly used for version control. Furthermore, releases should be tagged and archived to allow reproducing scientific results produced with a given release of a scientific software later on. Secondly, automatic and manual quality assurance procedures should be established in scientific software projects to minimize potential issues introduced when code changes. Software is nowadays highly complex and developers cannot foresee if a change may cause issues in code that depends on the modified function. Continuous integration systems can help by executing pre-defined automated tests that alert developers in case nightly builds fail because tests do not run or produce a change in outputs. Both version control and continuous integration are indisputable requirements for any software that aims to attract external contributions. Nevertheless, the best automated test system cannot replace manual testing, which should occur for critical core libraries or major releases to end users. Effort for such manual tests can be shared within the community. In our experience, power users of scientific software can be easily motivated to test a release candidate or beta version of a software because they enjoy exploring the newly introduced features and providing feedback to developers. For guiding testers during this process and to support developers who program tools that depend on a given software, release notes are highly recommended to inform the community about recent changes and what to take care of when upgrading to a newly released software version.

## Documentation

Software projects aiming to bring services and solutions to a broad audience require special emphasis on documentation: beyond a publication that mostly serves as an announcement of major software features, there are four kinds of modern software documentation: ^35^ 1) learning-oriented tutorials targeting beginners and introducing them to basic principles of a given software or software library; 2) goal oriented guides documenting typical use cases of a software, in which users can learn a new tool; 3) understanding-oriented discussions benefiting developers who might, especially in open-source projects, become contributors to extend and maintain a given software project; and 4) information-oriented reference manuals serving as a glossary of functionality, for example as a searchable database of individual functions to explore features and read their concise description.

Documentation should not be seen as the final step before launching a new software. It is rather a continuous process, particularly when new features are added. Furthermore, user feedback about the documentation can ultimately also lead to updates in the software. Software features that are intuitive to the developers might be complicated to explain in the user guide while writing the corresponding documentation, triggering a rethinking of how the feature is offered to the user and changing the GUI or the underlying algorithm. This will improve user experience and the documentation.

Finally, newly released bioimage analysis tools should always be provided with example datasets, for example included in the installer or uploaded on online platforms, such as Zenodo ( https://zenodo.org), chosen to illustrate some specific features. Additionally, complete protocols for processing these example data sets should be provided, for example via online platforms such as Protocols.io ( https://protocols.io). This decreases the likelihood that users trying a new tool for the first time will experience a crash or incomprehensible analysis result. Instead, running the desired analysis method on an example dataset will help them understand how others analyze data of similar kind to theirs.

## Interoperability

The term interoperability describes how different software can be combined in common workflows or substitute each other. Interoperability is mostly achieved through two levels: (i) external interoperability by the use of common file formats or libraries, and (ii) internal interoperability through a plugin mechanism. For constructing workflows efficiently, both internal and external interoperability have advantages and disadvantages: if possible, new software should adopt both to maximize interoperability.

If a software stores processing results in common file formats such as text (txt), comma-separated values (csv) or common image file formats, it can be considered as interoperable with other software that processes such files. Software that introduces unique, proprietary and/or closed-source file formats for handling data introduces a barrier and is not interoperable with other software. Much effort has been devoted by the bioimage community to promote external interoperability. For instance, Bio-Formats ^33^ is a software library for reading proprietary microscopy formats and converting to open standardized formats. These efforts are also resulting in community efforts towards new open formats. ^36, 37^

On a higher level, internal interoperability can be achieved by plugin mechanisms to ensure that within a given software application, modules from various origins can communicate with each other. Software such as ImageJ ^17^/Fiji, ^18^ CellProfiler, ^20^ KNIME, ^38^ or Icy ^19^ are known in the image data science field as platforms that are extensible using plugin architectures. Such interoperability can be beneficial to all software tools involved and drive innovation. A case in point is KNIME, which wanted to add image processing functionalities to its features set and decided against developing their own from scratch. Instead, they directly used ImageJ's functionalities and, in the process, helped improve both tools. ^39, 40^ Noticeably, an important project enabling interoperability is SciJava ( http://scijava.org) originally developed as part of the ImageJ ecosystem but then adopted by others; ^39^ it provides an overview of available Java libraries for scientific computing including file format import support and data visualization. ^21^

## Licensing

Licensing is an aspect of open-source software platforms that is often overlooked by developers in spite of its importance. More often than not, the license is eventually chosen when the source code is released, whereas it should be defined from the start. Licensing is essential because it defines how others are able to use the source code of a given project.

In most countries, selecting no license at all results in copyright by the author, all rights reserved, completely working against the very notion of open-source. This prevents code reusability because it would be unsafe to use copyrighted code.

A helpful resource in selecting a license is the Open Source Initiative ( https://opensource.org/), which maintains a list of approved open source licenses. Basically, they can be divided in three categories: strong copyleft, weak copyleft, and permissive licenses. Strong copyleft licenses (GPLv2, GPLv3, AGPL, etc.) grant permission to use, modify, and redistribute code *as long as* the original license remains intact for both the original project AND any project using any code of the original project. Although at first glance this sounds like the most open possible choice, there is a catch: a project with a strong copyleft license will force any subsequent project using part of its code to be under the same copyleft license and prevents the developer from using code from projects without this same license. In contrast, weak copyleft licenses (LGPL, MPL 2.0, etc.) do not extend their protection across linkage boundaries. This essentially means that, while the original project and its modifications will retain the weak copyleft license, any code linking to it will be allowed to use a different license (even proprietary code). Finally, permissive licenses (BSD, Apache 2.0, MIT, etc.) usually only retain the attribution restriction: any derived project will require statements giving credits to the original one while still being able to use, modify and distribute it, even commercially. Several of us felt quite protective of code at the beginning of our projects, thinking that a permissive license would risk our ability to maintain a unique grant-fundable project, but over time we have seen the benefits of our code bases being used to accelerate science however they are used by others.

## Publication

A project’s release may include executable software, code, documentation and tutorials; another milestone of an open-source software platform is its publication. For projects launched to solve a particular biological question, the best-case scenario is when the tool is ready to be released before the collaborators' biology study. In this case, a technical paper can present the tool and its new method without jeopardizing the biology publication; separate publication may even strengthen both.

Problems may arise when the biology paper is ready first and the software must be mentioned or described in it. Indeed, most journals are driven towards novelty, and presenting a tool that “only” provides a broader access to already published methods is often considered lacking novelty. This also holds true for plugins and is detrimental to putting efforts in making computational methods truly usable. And while several journals ^41^ may consider papers describing open-source software, managing to publish these tools in high-impact journals is highly challenging. Editors can struggle to predict the potential value and usability of a tool, especially for recent ones that have yet to reach a sizable audience. Nevertheless, we have detailed in this paper characteristics expected from a successful project; these criteria may help editors and reviewers in their assessments.

Academic labs mainly use short-term contracts. This implies that any successful open-source tool that managed to achieve long-term sustainability will hire several developers along the course of its life. As a consequence, one publication occurring at an early stage of a project will not accurately capture the whole picture of involved developers. Still, they all deserve recognition for their work and finding a way to reward them is essential. One solution is to write a new article with an updated list of authors when a new version of the project, with significant changes, is released. An ideal solution would be that the journal that first published the project would consider for peer-review this new paper, with criteria such as improved user-experience, new visualizations and methods added, etc.

Finally, two main indicators are, for better or worse, used by institutions and funding agencies to evaluate an open-source project: the reputation/impact factor of the journal in which the project is published and its number of citations. Therefore, properly citing bioimage analysis is of utmost importance for ensuring their long-term sustainability. For that reason, users should cite all tools used, whether for visualization, analysis or manipulation. When a plugin of a generic bioimage analysis platform is used, both the platform- and plugin-related papers should be cited. It is currently estimated that software tools are properly cited less than half of the time. ^42^

## Funding

Software maintenance and support is rarely glamorous, but is one of the most cost-effective ways to impact science: the efforts of a small number of individuals can dramatically improve the efficiency and accuracy of analysis performed by thousands of researchers, helping to maximize the value of data across many studies. However, traditional research funding agencies foster innovation, and thus, typically development of new methods and tools, as opposed to maintenance. This model arose and may have been sensible for technologies other than software, which are often adopted by companies after the proof-of-principle - developed with the research funding - is successfully demonstrated. Open-source software platforms are in a strategically suboptimal position when it comes to applying for funding in this context, because novelty is rarely the goal - indeed, robustness and standardization are usually more desirable. Thus, software maintenance and long-term support can often not be funded using research grants.

However, in recent years, exceptions have arisen: The German Research Foundation (DFG) offered funding specifically for “Research Software Sustainability”, ^43^ the U.S. Chan Zuckerberg Initiative (CZI) has begun supporting open-source software for science generally and for bioimaging specifically ^44^ and the National Institutes of Health (NIH) in the United States funded the Center for Open Bio-image Analysis (COBA) ( https://openbioimageanalysis.org/). Likewise, the European Cooperation in Science and Technology (COST) enabled forming the Network of European Bio-Image Analysts (NEUBIAS), which enables interdisciplinary exchange between developers and end-users on a high level leading to many collaborations and new projects in the context of open-source software for biological image data science. While the mentioned funding programs offered opportunities to scientific software-focused communities, it remains challenging as an individual research group to apply for funding to turn a software-focused research project into a community-driven open-source project which is maintained long-term and serves important needs of the scientific community. We would therefore advocate more funding agencies to follow the footsteps of CZI, NIH and DFG and create programs tailored for funding maintenance and continuous development of open source tools, since they have become crucial to most biology related research.

## Summary

In just a few decades, computational methods have radically transformed the way biological images are analyzed. From tedious manual quantification, life scientists now have access to an overwhelming array of automatic and unbiased analysis tools. In this context, open source software has played a key role, offering cutting-edge analysis methods to a wide audience. The value of open source is not simply that the software is typically free of financial cost: by disclosing the source code under a recognized license, open source software enables transparency, reproducibility, standardization, and a foundation that can be built upon by others. All these aspects are increasingly important as most funding agencies move towards Open Research.

Despite the great success achieved by many open source tools, developing new software from scratch should not be taken lightly. To prevent overlap with established tools, it is crucial to identify a major gap in the current landscape. The true costs of development and maintenance should be considered, and a careful long-term sustainability plan developed. If none of these conditions are met, we advocate for developing plugins to existing generic platforms, as this strongly alleviates both the maintenance issue, the activation barrier for users and the method’s overall accessibility.

If the strategy is still to develop a new tool, a few best practices should be implemented from the beginning. First, documentation should not be an afterthought, written at the time of the project’s release; it is a crucial ongoing process that should closely follow current developments. Second, version control should be embraced as it helps research reproducibility by allowing easy tagging of releases, brings intrinsic organization to a project and ensures that all contributions can be recognized. Third, any new project should select its license during its inception as it will have a profound impact on the way its code will be reusable. Fourth, GUI elements should be carefully designed to improve the project’s usability, when the audience suits it.

In spite of all these challenges, developing open source bioimage analysis software can be highly rewarding. The opportunity to durably impact a life science field by filling an analysis gap, and therefore to reach a whole new community, is a worthy motivation. Unfortunately, even the most successful open source tools still struggle to obtain funding for maintenance and continuous development. There is a clear mismatch between these difficulties and the influence of those tools that help thousands of life scientists shaping their data into quantitative measures. For that reason, we hope that funding open source tools will become easier in the future.

## Data availability

No data is associated with this article.
