# Supplementary material for: Developing open-source software for bioimage analysis: opportunities and challenges
Source: F1000Res. 2021 Apr 19;10:302. [Version 1] doi: 10.12688/f1000research.52531.1 (PMC8226416; doi:10.12688/f1000research.52531.1)
Supplement: Peer review history

# Peer review: Reviewer response for version 1

**DOI:** 10.5256/f1000research.55826.r87725

**Published:** 2021-06-24

**Authors:** Nico Stuurman

**Affiliations:** 1 Department of Cellular and Molecular Pharmacology, Howard Hughes Medical Institute, University of California, San Francisco, San Francisco, CA, USA

This manuscript, written by leaders in the field, provides a wonderful overview of the considerations, pitfalls, and challenges for developers of open source software for bioimage analysis. It is of interest to users, who will get a taste of the efforts behind the tools they are using, and to (aspiring) developers, who are provided with a great overview of all aspects of tool development, helping them make sound choices in their work. The manuscript is well organized, well written, and frankly, a pleasure to read. There are a few more subjects/questions related to bioimage analysis software development that I am interested in, and would love to see the authors’ take:

- The relation to commercial software. Back a few decades ago, algorithms were developed in academia, and User Interfaces to work with these algorithms were created by commercial companies (NIH Image / ImageJ was one of the first software packages to break this “tradition”, and received significant push back from commercial entities). I would love to see a description of the current relation between open source and commercial bioimaging software, as well as a description what this relation ideally would be.
- Perhaps related and perhaps partly covered: the tension between creating new approaches to problems for which solutions exist (open source or not), versus using/embracing/extending existing solutions. The software development world in general has adopted the strategy to try many approaches and let the most widely used or durable solution(s) stand. Due to the limited resources in the bioimage software world, this hardly seems the most optimal approach, yet many are tempted to make a better solution than the one that already exists.
- Distribution: Code signing / security. Operating systems and IT infrastructures are increasing security requirements for desktop software, and a future where unsigned/non-confirming software can no longer be executed seems near, yet there are few (I don’t remember encountering any) one click installers of bioimage analysis software that are signed, ImageJ needs moving the executable out of and back into the Applications folder to get it to run on modern OSX, and Fiji advices against installing into the standard “Program Files” directory on Windows. I am not sure whether this is a minor financial and technical hurdle to be addressed by individual developers or something that needs more community attention.

Again, the manuscript is great as is, and would totally understand if the authors feel these issues are out of scope.

Is the topic of the opinion article discussed accurately in the context of the current literature?

Yes

Are arguments sufficiently supported by evidence from the published literature?

Yes

Are all factual statements correct and adequately supported by citations?

Yes

Are the conclusions drawn balanced and justified on the basis of the presented arguments?

Yes

Reviewer Expertise:

Cell Biology, single molecule imaging, open source software for microscope control.

I confirm that I have read this submission and believe that I have an appropriate level of expertise to confirm that it is of an acceptable scientific standard.

# Peer review: Reviewer response for version 1

**DOI:** 10.5256/f1000research.55826.r83552

**Published:** 2021-05-04

**Authors:** Loïc A. Royer

**Affiliations:** 1 Chan Zuckerberg Biohub, San Francisco, CA, USA

The opinion article "Developing open-source software for bioimage analysis: opportunities and challenge." addresses the important topic of developing software for bioimage analysis. 

 The authors discuss all aspects including the genesis of a project, the choice of scope and audience, technical choices, the role of communities, the ever important documentation, licensing, publication, and the crucial aspect of long-term funding and maintenance.

 I wholeheartedly agree with everything said here. Nothing here is controversial or in my opinion subject to debate. The issue perhaps is that the bits of wisdom in this piece should be more broadly shared, and in that sense I welcome its publication. If debate must arise, let the community as a whole do the debating.

 Here comes some constructive feedback to improve the manuscript: I find the first figure a bit cryptic, the different symbols are nice but there is no legend, and they are combined in evocative but unclear ways. It takes a bit too long to 'decode' the figure in my honest opinion. I would rethink the graphical design, make things more explicit, and add a bit of text to guide the reader.

Is the topic of the opinion article discussed accurately in the context of the current literature?

Yes

Are arguments sufficiently supported by evidence from the published literature?

Yes

Are all factual statements correct and adequately supported by citations?

Yes

Are the conclusions drawn balanced and justified on the basis of the presented arguments?

Yes

Reviewer Expertise:

bioimage analysis, microscopy, biology, algorithms,  optics,

I confirm that I have read this submission and believe that I have an appropriate level of expertise to confirm that it is of an acceptable scientific standard.
